# Underreporting of Adverse Events Following COVID-19 Vaccination Among Healthcare Professionals in Poland: Potential Implications for Vaccine Hesitancy

**DOI:** 10.3390/tropicalmed10110320

**Published:** 2025-11-13

**Authors:** Jakub Grabowski, Anna Niebrzydowska, Aleksandra Brzozowska, Przemysław Waszak, Paweł Zagożdżon, Shan Ali, Tomasz Brancewicz, Monika Wolff, Aleksandra Macul-Sanewska, Leszek Bidzan

**Affiliations:** 1 Division of Developmental, Psychotic and Geriatric Psychiatry, Department of Psychiatry, Faculty of Medicine, Medical University of Gdańsk, 80-282 Gdańsk, Poland; 2 Regional Psychiatric Hospital, 80-282 Gdańsk, Poland; 3 Adult Psychiatry Scientific Circle, Division of Developmental, Psychotic and Geriatric Psychiatry, Department of Psychiatry, Faculty of Medicine, Medical University of Gdańsk, 80-282 Gdańsk, Poland; 4 Department of Hygiene and Epidemiology, Faculty of Medicine, Medical University of Gdańsk, 80-210 Gdańsk, Poland; p.waszak@gumed.edu.pl (P.W.);; 5 Neurology Department, Mayo Clinic, Jacksonville, FL 32224, USA; 6 Optimmed Mental Health Centre, 80-767 Gdańsk, Poland; 7 Department of Health Sciences, Pomeranian University in Słupsk, 76-200 Słupsk, Poland

**Keywords:** COVID-19, COVID-19 vaccine, vaccine hesitancy, adverse events following immunization, vaccine side effects, vaccine attitude

## Abstract

This study aimed to assess the prevalence and reporting rate of adverse events following immunization (AEFIs) among healthcare professionals (HCPs) and students of health-related disciplines after COVID-19 vaccination. It was conducted at the beginning of the vaccination campaign in Poland (February 2021), when vaccines were only available to limited groups of recipients, mainly those related to healthcare. Questionnaires were distributed among HCPs in the Pomeranian voivodeship (N = 1063) and students at the Medical University of Gdańsk (N = 1506). The primary objective was to compare respondents’ self-reported AEFI notifications with official reports published by the National Sanitary Inspectorate. A total of 240 participants declared having reported at least one AEFI, whereas official reports from the same period indicated that only 194 individuals had reported AEFIs in the entire voivodeship. This translates into significant differences in notification rates (14.9% and 0.09%, respectively). A detailed breakdown into local and systemic AEFIs also revealed significant discrepancies with official reports (850 vs. 329 and 1137 vs. 46, respectively). The most common reasons for not reporting were managing the symptoms on one’s own and perceiving the symptoms as not severe enough to report. Underreporting of AEFIs is an issue that requires attention from both the scientific community and public health authorities, as it may hinder reliable vaccine safety assessment and contribute to increased vaccine hesitancy.

## 1. Introduction

Severe acute respiratory syndrome coronavirus 2 (SARS-CoV-2) is an RNA virus that primarily attacks the respiratory system, causing coronavirus disease 2019 (COVID-19). The first cases were reported in China’s Wuhan province in November 2019, while the global pandemic was proclaimed by the World Health Organization (WHO) in March 2020 [1,2]. In response to the rapidly increasing incidence of the disease, measures were quickly undertaken to develop a vaccine [3,4,5,6]. The first approved vaccine in the European Union was the mRNA vaccine BNT162b2 by Pfizer and BioNTech (authorized by the European Commission decision of 21 December 2020). Two further vaccines were authorized in January 2021: mRNA-1273 by Moderna and the vector vaccine AZD1222 by Oxford-AstraZeneca [7].

In Poland, the vaccination campaign began at the end of December 2020 and was initially aimed at healthcare professionals (HCPs) (so-called group zero), such as physicians, nurses, midwives, paramedics, other employees of healthcare entities, pharmacists, medical university employees, and medical students [8]. A total of 3,336,354 doses were distributed in Poland until the end of February 2021 (of which 216,850 were in the Pomeranian voivodeship) [9]. At that time, the BNT162b2 vaccine was the most frequently administered, especially among HCPs.

The current literature considers BNT162b2 a safe vaccine with only short-term, mild-to-moderate symptoms, with a low incidence of serious adverse events following immunization (AEFIs). AEFIs are defined by the WHO as any undesirable medical occurrences that happen after vaccination but are not necessarily caused by it [9]. Such events may involve any unfavorable or unintended sign, abnormal laboratory result, symptoms, or illness. AEFIs may be related to the vaccine itself, the vaccination procedure, immunization anxiety, or be caused by a coincidental factor [9]. There is no consensus on the time window within which an event must occur to be classified as an AEFI. The WHO uses a broad definition without any specified time window. However, it emphasizes that the timing of an AEFI may be crucial in establishing a causal relationship [10]. The Centers for Disease Control (CDC) uses a list of reportable events following vaccination with specific time intervals for different vaccines and their AEFIs [11]. The most common AEFIs that were reported after the BNT162b2 vaccine included injection site pain, fatigue, muscle pain, local swelling, joint pain, headache, fever, chills, and nausea [12,13,14,15,16,17].

Polish legal regulations require a physician to report a suspected AEFI to a local sanitary and epidemiological station within 24 h. AEFIs can also be reported individually by the person concerned to the Office for Registration of Medicinal Products, Medical Devices and Biocidal Products [18]. All AEFIs are then collected by the National Sanitary Inspectorate, which presents official reports. COVID-19 vaccines have come under additional monitoring from the European Medicines Agency (EMA) [19], which makes the reporting of AEFIs particularly important in terms of measuring and ensuring the safety of the preparation.

As with many other infectious diseases, vaccine hesitancy emerged as a major challenge during the COVID-19 pandemic, hindering efforts to achieve optimal vaccination coverage and control of the pandemic [20,21,22,23]. In many countries, the introduction of COVID-19 vaccines was met with considerable resistance and negative reactions from a large part of the public [24,25,26,27,28,29]. Common arguments raised by opponents of vaccination included the accelerated process of vaccine development and registration, side effects, and insufficient information [29,30,31,32,33,34,35]. Notably, knowledge, perceived risk, and trust are recognized as key factors influencing vaccination decisions [36,37]. Since post-marketing safety surveillance relies heavily on reporting of AEFIs, accurate and timely reporting plays an important role in maintaining public trust and mitigating vaccine hesitancy [38].

Pharmacovigilance studies have shown that AEFIs are often underreported, which may affect the assessment of vaccine safety in the population [39,40,41,42]. A similar pattern is well-documented for adverse drug reactions (ADRs). Underreporting of ADRs by HCPs is well-documented [43,44,45,46], yet little research has focused on underreporting of AEFIs by HCPs. In this study, we aimed to independently check the rate of reporting AEFIs by HCPs and students of health-related disciplines in Poland after they themselves received the COVID-19 vaccination and to discuss these findings in the broader context of vaccine hesitancy.

## 2. Materials and Methods

The research was conducted between 2 February 2021 and 25 February 2021 during the initial phase of the COVID-19 vaccination campaign in Poland. Data was collected through an online questionnaire distributed via institutional mailing lists of healthcare facilities, the internal mailing list of the Medical University of Gdańsk (MUG), and closed social media groups dedicated to HCPs in the Pomeranian Voivodeship. The study population included physicians, dentists, nurses, midwives, paramedics, laboratory diagnosticians, physical therapists, pharmacists, clinical psychologists, and students of health-related disciplines at MUG. Participation was voluntary and anonymous. Informed written consent was obtained electronically from all participants before survey initiation. The study was conducted according to the guidelines of the Declaration of Helsinki and was approved by the Institutional Ethics Committee of the Medical University of Gdańsk (NKBBN/153/2021).

The questionnaire was developed in Polish by the research team. It included closed questions (some with the option of multiple choices and adding an own answer). The initial part of the questionnaire included demographic data (gender, age, occupation, field of study—in case of students). The next sections of the questionnaire dealt with the type of vaccine received, the occurrence and type of local and/or systemic AEFIs, and the question of officially reporting AEFIs. We quantified whether people reported their AEFIs officially and prompted respondents to list the reasons for not reporting AEFIs. Respondents were also asked whether they used any medications to prevent or alleviate AEFIs. Self-reported data on AEFIs and reporting behavior were compared with publicly available national pharmacovigilance reports published by the National Sanitary Inspectorate. All data were curated in Microsoft Excel, and analyses were performed in Python 3.11.5 (pandas, NumPy, SciPy, statsmodels) with GPT-5 Thinking-assisted code. Associations between categorical variables were assessed using the chi-squared (*χ*^2^) test. Differences between continuous variables were evaluated with Welch’s *t*-test. When multiple groups were compared (e.g., professions or study programs), pairwise post hoc tests were performed within the logistic model (Wald contrasts), and the resulting *p*-values were adjusted for multiple testing using the Benjamini–Hochberg false discovery rate. A *p*-value < 0.05 was considered statistically significant.

## 3. Results

A total of 2569 people completed the survey, of whom 1939 were females and 630 were males. Respondents were between 18 and 78 years old (mean = 29.3, SD = 11.7). Students were significantly younger than other respondents (22.2 ± 2.4 vs. 39.5 ± 12.2 years; *p* < 0.001). The largest professional group consisted of students (58.6%), followed by physicians (16.0%), nurses (4.0%), and pharmacists (2.8%). Among students (N = 1506), the most common fields of study were medicine (52.7%), nursing (17.3%), pharmacy (13.4%), and psychology (8.5%), with smaller proportions representing physiotherapy, public health, and other disciplines. Baseline demographic characteristics are presented in Table 1 and Table 2.

### 3.1. Prevalence and Notification Rates of AEFIs

A total of 680 respondents received one dose of the vaccine, and 1889 received two doses. The BNT162b2 vaccine was administered to 2529 respondents (98.3%), mRNA-1273 to 28 respondents (1.1%), and AZD1222 to seven respondents (0.3%). The remaining 0.3% of respondents did not remember the name of the vaccine. A total of 1616 respondents declared to have experienced at least one AEFI after at least one dose of the COVID-19 vaccine. A total prevalence of AEFIs was calculated as the number of people who declared any AEFI after any dose of vaccine vs. the number of people who received any dose of vaccine, amounting to 62.9%.

As for reporting AEFIs, 240 respondents claimed to have performed it, resulting in a declared notification rate of 14.9%. The notification rate was calculated as the number of people who claimed to have officially reported an AEFI vs. the number of people who declared any AEFI after any dose of vaccine.

Notification rates differed between sexes, professions, and student courses. Females claimed to have reported AEFIs significantly more often than males (16.0% vs. 11.3%; *p* = 0.022) (Table 3).

Notification rates were compared across professions, with “Other” professions fully excluded from the analyses. Global differences across professions were statistically significant (*p* = 0.0148). However, no pairwise differences remained significant after FDR correction. Table 4 presents notification rates by profession.

Overall, 945 students (62.7%) declared AEFIs, and 149 (15.8%) claimed to have reported them officially. There were no significant differences in AEFI notification rates between study courses. The overall test was not significant (*p* = 0.389), and no pairwise comparisons were significant after multiple-testing correction (Table 5).

AEFIs were significantly more prevalent among respondents with prior COVID-19 (confirmed by a positive test result) than among those without prior COVID-19 infection (68.6% vs. 62.2%; *p* = 0.038), but the notification rates did not differ significantly between the two groups (16.3% vs. 14.7%; *p* = 0.546) (Table 6).

### 3.2. Comparison with Official Sanitary Reports

Significant discrepancies between respondents’ declarations on AEFI reporting and official reports of the National Sanitary Inspectorate could be observed at all stages of the study, which is presented in detail in Table 7. At the endpoint of the study (i.e., 25 February 2021), out of 2,990,683 vaccinations performed in Poland and 204,953 vaccinations performed in the Pomeranian voivodeship, official reports confirmed only 3103 cases of AEFIs nationwide and 194 cases of AEFIs in the Pomeranian voivodeship [47], giving a notification rate of 0.1% and 0.09%, respectively. Respondents who claimed to have reported an AEFI exceeded the number of AEFIs reported in the entire voivodeship, which may suggest reporting inconsistencies. Please note that the data presented in the subsequent rows of the table are cumulative.

### 3.3. Analysis of Local and Systemic AEFIs

After that, a detailed analysis of local and systemic AEFIs was performed. AEFIs for the first and second doses were calculated separately and then added. In absolute figures, the total number of AEFIs declared by respondents was equal to 10,435, of which 5133 were local AEFIs and 5302 were systemic AEFIs. Officially, only 440 AEFIs were reported, of which 337 were local and 103 were systemic. After subtracting the AEFI categories not mentioned by respondents but included in the reports (e.g., fainting, stroke, diarrhea, numbness of the tongue), the numbers of reported local and systemic AEFIs amounted to 329 and 46, respectively. In all categories analyzed, there were evident inconsistencies between respondents’ statements and official reports. A detailed comparison is presented in Table 8 and Table 9. According to official reports of the National Sanitary Inspectorate, most cases were mild and involved mainly pain and redness at the injection site [47].

### 3.4. Reasons for Underreporting AEFIs

As mentioned in the Introduction, the problem of underreporting adverse events is critical; therefore, an attempt has been made to understand its causes in the context of COVID-19 vaccines. The most popular reasons for not reporting the AEFIs were managing the symptoms themselves and not needing help (N = 811) and perceiving the symptom as not severe enough to report (N = 791). A total of 403 people did not have sufficient knowledge of how to report an AEFI, including 52 physicians, four dentists, six pharmacists, 168 medical students, seven dentistry students, and two pharmacy students. Some respondents claimed that an AEFI does not need to be reported if it is already listed in the information leaflet.

One of the strategies to prevent or manage undesirable symptoms related to vaccination was taking medications. This strategy was implemented by 929 respondents (i.e., 36.2% of all respondents), 75.5% of whom (N = 701) were the people who declared AEFIs but did not report them. Taking medications was popular, especially among nurses and students of the following disciplines: medicine, nursing, midwifery, pharmacy, and electroradiology. In each of these groups, at least 40% of people were taking medications before and/or after the vaccination.

## 4. Discussion

The obtained results regarding the overall prevalence and profile of adverse events following COVID-19 vaccination in HCPs are consistent with many previous studies [48,49,50,51,52,53]. The strength of this study was the attempt to objectively assess the extent of underreporting. A study by Noh et al. [39] was similar in concept and design, but it did not compare respondents’ declarations with official epidemiological reports and was not specifically targeted at HCPs. Similarly, Sankar et al. indirectly demonstrated underreporting of AEFIs by comparing reports from South Africa with those of other countries [52]. In the study by Sauserienė et al., the incidence of AEFIs among HCPs was estimated at 53.6%, the majority of which were local and mild. Although the authors did not objectify the notification rate in the probe, they pointed out that it was only 0.27% nationwide at that time [54]. Jeśkowiak et al., who adopted a very similar method of analysis to ours, suggest significant underreporting among HCPs in Poland. In their study, 4.6% of respondents (out of 80% who experienced AEFIs) claimed to have reported to the National Sanitary Inspectorate [55]. Noteworthy is the slightly different profile of respondents in our sample, with a higher proportion of physicians and fewer pharmacists.

Underreporting of AEFIs may indirectly increase vaccine hesitancy by undermining trust and the sense of transparency in the surveillance system. Polish official communications repeatedly emphasized that AEFIs were “extremely rare,” with the Minister of Health publicly quoting 37 AEFIs after more than 250,000 doses on 12 January 2021 while introducing a compensation scheme [56]. Our data suggest that, even in a highly health-literate cohort, only a minority of those experiencing AEFIs reported them through official channels, indicating that low administrative counts likely reflected underreporting rather than absence of events. In the absence of accurate AEFI reporting, information gaps may often be filled with anecdotal evidence or misinformation on social media, which may amplify fears and doubts [57,58,59,60,61,62,63]. Conversely, transparent negative communication about risks may temporarily lower acceptance but permanently increase trust in health authorities [63]. Such transparency is crucial in a time when anti-vaccination views are on the rise—a movement that is predicted to dominate pro-vaccination in a decade [64,65,66].

COVID-19 vaccines have been a key factor in reducing infection rates, disease severity, hospitalizations, and mortality [67,68,69]. Early estimates suggested that a vaccination coverage of at least 60–70% is needed to control viral transmission [70,71,72,73,74], which was in line with the WHO’s imperative for 70% vaccination coverage worldwide by mid-2022 [75]. Nevertheless, global vaccination coverage (defined as the percentage of the total population vaccinated with at least one dose) remained highly variable. Within the European Union, the cumulative vaccine uptake reached 75.6%, with substantial disparities between countries. In Poland, 60.8% of the population has been vaccinated with at least one dose [76].

A meta-analysis by Roy et al. identified safety concerns and potential side effects as the most frequently cited factors influencing COVID-19 vaccine acceptance or hesitancy [77]. Kumar et al. described four primary drivers of hesitancy towards COVID-19 and influenza vaccines: concerns over safety, lack of trust, perceived lack of necessity, and cultural beliefs [78]. Also noteworthy is the concept proposed by Perrone et al., in which the key driver of COVID-19 vaccine hesitancy is a lack of control. It is further related to distrust of the government, confusing communication, lack of confidence in the validity and security of the vaccine, and personal health issues [57]. Numerous studies conducted in the Global South countries point to various reasons for vaccine hesitancy, including distrust of authorities, lack of knowledge about the vaccine, misconceptions about the risk of infection, cultural and religious influences, socioeconomic factors, and low levels of trust in the healthcare system [79,80,81,82].

With the release of subsequent boosters, there is a noticeable decline in vaccination coverage [76,83,84,85]. Negative experiences with vaccines appear to be a significant factor influencing the willingness to accept a booster, which is noteworthy given the high incidence of AEFIs [86,87,88]. This can possibly apply to HCPs as well, with AEFIs experienced with previous doses and regretting the decision to get vaccinated acting as factors reducing the willingness to receive a booster in the cohort [88,89]. Following the Omicron wave, there is also noticeable decoupling between official recommendations and vaccination coverage in many countries [90]. Interesting conclusions come from a survey conducted among pharmacists in Pakistan. A significant proportion of respondents believed that pharmaceutical companies were withholding some information about the vaccine to increase sales [91].

Negative attitudes towards vaccination have been documented since the late 19th century, but in recent years, they have intensified significantly, prompting the WHO to classify vaccine hesitancy as one of the 10 major public health problems in 2019 [92,93]. This is reflected in declining vaccination coverage for childhood diseases, particularly measles and pertussis. In the European Union, only four countries (Hungary, Malta, Portugal, and Slovakia) currently achieve the 95% vaccination coverage required to maintain herd immunity against measles [94]. In the United States of America and the United Kingdom, coverage is also below this threshold (90.8% and 88.9%, respectively) [95,96]. This corresponds with an increasing incidence of measles, with over 127,000 cases in the WHO European Region in 2024, the highest number in 25 years [97]. In the United States, the number of cases confirmed by August 2025 has already exceeded that of 2019 and is the highest since 2000, when the disease was declared eliminated [98]. We would like to remind the medical community that reliable and widespread AEFI reporting is indeed crucial to find potentially serious adverse effects, an example of which was the identification of a higher risk of narcolepsy in individuals inoculated with the H1N1 influenza vaccine [99]. We believe that the benefits of immunization outweigh the side effects; however, understanding the frequency and viability of AEFIs may help populations become more convinced about vaccination. Accurate AEFI reporting was requested by both the WHO and local institutions [9,100,101,102,103,104].

The current research also shows that some respondents were not telling the truth about reporting AEFIs to the National Sanitary Inspectorate. A possible explanation is the search for social approval. Having appropriate professional competencies, HCPs may be expected to report reliably and take responsibility for public health issues, especially in such special circumstances as the pandemic.

What should be considered is that interviewing healthcare workers poses an inherent risk of bias in AEFI reporting. Knowledge and understanding of medical issues might lead to disregarding symptoms and underreporting AEFIs, to which the general population would probably pay more attention. This is confirmed by respondents’ answers on the reasons for not reporting AEFIs. Medical knowledge may be the reason for their eagerness to cope with symptoms on their own and their belief that symptoms were not serious enough to report. A more complex view is presented by García-Abeijon et al. [105], who identified the following reasons for underreporting: ignorance (a belief that only serious ADRs should be reported), lethargy (e.g., procrastination), complacency (a belief that a preparation must be safe if it is on the market), diffidence (fear of opinion of the rest of medical community), and insecurity (a belief that it is impossible to determine with certainty whether a drug is responsible for a given symptom). Secondly, there is obviously a self-selection bias, as HCPs generally had a more positive outlook on vaccines [106,107], which might have influenced their decision-making process for reporting any negative findings, such as AEFIs. The sample may overrepresent HCPs with a greater interest in vaccination and safety issues, which may reduce the generalizability of the data. This could be partially verified by comparing the incidence of particular AEFIs in the study group with official data from clinical trials at that time [108]. For example, pain at the injection site was reported less frequently by study participants than in the manufacturer’s information (59.1% vs. 83.1%). Fatigue (49.2% vs. 47.4%) and headache (38.5% vs. 41.9%) had comparable frequencies. However, many AEFIs, including fever, muscle pain, joint pain, and swelling at the injection site, were reported significantly more often by HCPs participating in the study. The high declared prevalence of AEFIs in the survey may also be the consequence of online data collection. In such circumstances, respondents may feel more comfortable and take their time to reflect on their experiences. Tools that allow patients to report adverse reactions online are considered important to enhance safety and allow better pharmacovigilance [109,110,111].

It should be noted that some respondents decided to take medications to prevent or ease symptoms related to vaccination. There are also reports of medics with a personal history of severe allergic reactions who, for fear of COVID-19, decided to take antihistamines to prevent anaphylaxis or hypersensitivity and not to be disqualified from the vaccination [112].

A problem that emerges from the study is also a lack of knowledge of some physicians, dentists, pharmacists, and students about how to report an AEFI, which may have impacted the underreporting rate. However, such information was easily accessible at governmental websites at that time, as well as in information leaflets that were distributed in vaccination centers. As no prerequisite formal knowledge was needed to report an AEFI, with little effort, they could easily perform it. A more serious issue, however, is the misconception that there is no need to report side effects if they have already been described in the leaflet, especially since the vaccine was subject to specific EMA monitoring, which was indicated in the leaflet by a black triangle.

The main limitation of the study was variability in the timing of questionnaire completion (ranging from a few days to several weeks post-vaccination), which may have affected recall accuracy. Respondents completing the survey soon after vaccination may not yet have experienced all AEFIs, while those completing it later may have forgotten some.

## 5. Conclusions

This study offers a new perspective related to the underreporting of AEFIs. Our findings indicate that HCPs fail to report a large proportion of AEFIs after their own vaccination. In the study sample (with no significant differences between individual professions), there was a significant difference between the declared prevalence and notification rates of AEFIs. In addition, when compared with sanitary reports, a significant discrepancy was found in reporting specific AEFIs.

Underreporting of AEFIs among HCPs presents a serious challenge, as it may contribute to vaccine hesitancy by undermining trust in health institutions and the transparency of the immunization. Preventing vaccine hesitancy should remain a public health priority in the post-pandemic era, as COVID-19 becomes endemic yet continues to threaten vulnerable populations. At the international level, it is important to ensure that reporting standards are harmonized across countries where a given vaccine is authorized, as this may facilitate reliable data analysis and support the development of consistent, evidence-based recommendations. Strengthening AEFI reporting systems supports transparent safety monitoring, maintains public confidence, and helps ensure effective responses to possible future outbreaks. Reporting may be increased when the information is submitted online rather than in person. Thus, we encourage vaccination facilities to establish internet AEFI reporting, as in our study.

In the national context, reliable and efficient systems for analyzing AEFI data and accurate communication about possible AEFIs appear to be important. Accurate reporting can help prepare patients for potential AEFIs, allowing them to feel informed and safe. Awareness of common, generally mild post-vaccination symptoms (e.g., transient headache) can reduce unnecessary primary care visits, thereby alleviating pressure on already overburdened healthcare systems.

The study results also suggest possible knowledge gaps or misunderstanding of AEFIs by HCPs. Thus, more attention should be paid to the education of physicians, dentists, and pharmacists on AEFI reporting, alongside measures to simplify the reporting process. Training in this area should be included in the study program and continuing education of HCPs.

Although conducted in Poland, these findings are likely relevant to other countries that were vaccinating their populations at the same time, with the same vaccine and with similar AEFI reporting regulations. Further research should explore physicians’ attitudes towards AEFI reporting and identify practical solutions to improve pharmacovigilance in vaccination programs.

## Figures and Tables

**Table 1:** Demographic characteristics of the study participants (N = 2569).

Characteristic	Group of Respondents	N	%	Age (Mean ± SD)
Sex	Female	1939	75.5	29.2 ± 11.6
	Male	630	24.5	29.9 ± 12.0
Profession	Student	1506	58.6	22.2 ± 2.4
	Physician	411	16.0	41.1 ± 12.7
	Dentist	39	1.5	32.7 ± 10.7
	Nurse	102	4.0	39.8 ± 12.1
	Midwife	32	1.3	29.2 ± 11.1
	Paramedic	2	0.1	40.5 ± 19.1
	Laboratory diagnostician	64	2.5	37.8 ± 11.7
	Physical therapist	28	1.1	30.0 ± 7.6
	Pharmacist	71	2.8	35.0 ± 10.1
	Clinical psychologist	31	1.2	44.2 ± 10.6
	Other *	283	11.0	41.0 ± 11.1

N—number, SD—standard deviation. * Respondents of other professions related to healthcare who did not fall into any category listed in the questionnaire (e.g., dietitians, administrative employees, university lecturers, physician assistants).

**Table 2:** Demographic characteristics of the study participants—students (N = 1506).

Course	N	%	Age (Mean ± SD)
Medicine PL Division	778	51.7	22.4 ± 2.3
Medicine ENG Division	2	0.1	27.0 ± 1.4
Dentistry	100	6.6	23.0 ± 3.2
Nursing	87	5.8	20.8 ± 1.3
Midwifery	37	2.5	20.7 ± 1.3
Pharmacy	160	10.6	22.4 ± 2.0
Physical therapy	53	3.5	21.6 ± 3.2
Medical analytics	67	4.4	21.4 ± 1.5
Paramedics	18	1.2	21.4 ± 1.7
Health psychology	50	3.3	21.6 ± 1.9
Electroradiology	28	1.9	20.7 ± 1.1
Dental techniques	23	1.5	20.6 ± 2.3
Dietetics	48	3.2	22.0 ± 2.7
Public health	20	1.3	23.4 ± 6.5
Environmental health	17	1.1	20.8 ± 1.2
Pharmaceutical and cosmetic industry	18	1.2	23.8 ± 1.4

N—number, SD—standard deviation, PL Division—Polish Division, ENG Division—English Division.

**Table 3:** Number of declared and allegedly reported AEFIs; notification rates by sex.

Sex	With AEFI (N)	Reported (N)	Notification Rate (%) (95% CI)
female	1219	195	16.0 (14.0–18.2)
male	397	45	11.3 (8.6–14.8)

N—number, AEFI—adverse event following immunization, CI—confidence interval.

**Table 4:** Number of declared and allegedly reported AEFIs; notification rates by profession.

Profession	With AEFI (N)	Reported (N)	Notification Rate % (95% CI)
Student	945	149	15.8 (13.6–18.2)
Physician	288	28	9.7 (6.8–13.7)
Dentist	17	1	5.9 (1.0–27.0)
Nurse	57	9	15.8 (8.5–27.4)
Midwife	19	7	36.8 (19.1–59.0)
Paramedic	1	0	0.0
Laboratory diagnostician	40	6	15.0 (7.1–29.1)
Physical therapist	16	3	18.8 (6.6–43.0)
Pharmacist	54	7	13.0 (6.4–24.4)
Clinical psychologist	20	6	30.0 (14.5–51.9)
Other *	159	24	15.1 (10.4–21.5)

N—number, AEFI—adverse event following immunization, CI—confidence interval. * Respondents of other professions related to healthcare who did not fall into any category listed in the questionnaire (e.g., dietitians, administrative employees, university lecturers, physician assistants).

**Table 5:** Number of declared and allegedly reported AEFIs; notification rates by study course.

Study Course	With AEFI (N)	Reported (N)	Notification Rate % (95% CI)
Medicine PL Division	537	81	15.1 (12.2–18.4)
Medicine ENG Division	2	0	0.0
Dentistry	68	10	14.7 (7.3–25.4)
Nursing	48	8	16.7 (7.5–30.2)
Midwifery	18	3	16.7 (3.6–41.4)
Pharmacy	95	15	15.8 (9.1–24.7)
Physical therapy	32	10	31.2 (16.1–50.0)
Medical analytics	32	4	12.5 (3.5–29.0)
Paramedics	6	1	16.7 (0.4–64.1)
Health psychology	31	3	9.7 (2.0–25.8)
Electroradiology	13	1	7.7 (0.2–36.0)
Dental techniques	11	3	27.3 (6.0–61.0)
Dietetics	27	7	25.9 (11.1–46.3)
Public health	11	0	0.0 (0.0–28.5)
Environmental health	10	3	30.0 (6.7–65.2)
Pharmaceutical and cosmetic industry	4	0	0.0

N—number, AEFI—adverse event following immunization, PL Division—Polish Division, ENG Division—English Division, CI—confidence interval.

**Table 6:** Number of declared and allegedly reported AEFIs; notification rates by prior COVID-19 status.

Prior COVID-19	With AEFI (N)	AEFI Prevalence % (95% CI)	Reported (N)	Notification Rate % (95% CI)
Yes	190	68.6 (62.9–73.8)	31	16.3 (11.7–22.2)
No	1426	62.2 (60.2–64.2)	209	14.7 (12.9–16.6)

N—number, AEFI—adverse event following immunization, CI—confidence interval.

**Table 7:** Comparison of the number of AEFIs cases allegedly reported by respondents with official reports of the National Sanitary Inspectorate for the Pomeranian voivodeship [47].

Date	According to Respondents	According to Reports
2 February 2021	102	69
3 February 2021	183	80
4 February 2021	197	80
10 February 2021	222	103
11 February 2021	224	113
25 February 2021	240	194

**Table 8:** Local AEFIs—comparison of the number of AEFIs allegedly reported by respondents with reports of the National Sanitary Inspectorate (cumulative data from dose one and dose two).

AEFI	No. of Declared Cases	No. of Allegedly Reported Cases	No. of Cases in Reports
pain at the injection site	2620	408	161
redness at the injection site	362	64	158
itching at the injection site	185	30	0
swelling at the injection site	487	100	2
rash at the injection site	14	4	3
urticaria at the injection site	25	3	1
limb pain	1438	241	2
acute peripheral facial palsy	2	0	2
**Total local AEFIs**	**5133**	**850**	**329**

AEFI—adverse event following immunization.

**Table 9:** Systemic AEFIs—comparison of the number of AEFIs allegedly reported by respondents with official reports of the National Sanitary Inspectorate (cumulative data from dose one and dose two).

AEFI	No. of Declared Cases	No. of Allegedly Reported Cases	No. of Cases in Reports
fatigue	1346	287	0
chills	561	117	5
fever	587	135	10
arthralgia	459	99	4
myalgia	804	171	6
nausea	244	45	1
sleeplessness	176	43	0
head pain	924	204	6
lymphadenopathy	101	13	4
anaphylaxis	1	0	2
oversensitivity	99	23	8
**Total systemic AEFIs**	**5302**	**1137**	**46**

AEFI—adverse event following immunization.

## Data Availability

The raw data supporting the conclusions of this article will be made available by the authors upon request. The manuscript includes references to reports from the National Sanitary Inspectorate that were downloaded by the authors in February 2021 and (due to their current unavailability online) placed in a public Figshare repository ([47]).

## Abbreviations

The following abbreviations are used in this manuscript:

AEFI	adverse event following immunization
COVID-19	coronavirus disease 2019
HCP	healthcare professional
SARS-CoV-2	severe acute respiratory syndrome coronavirus 2
WHO	World Health Organization
CDC	Centers for Disease Control
ADR	adverse drug reaction
EMA	European Medicines Agency
MUG	Medical University of Gdańsk
N	number
